# The Physician1Presidential address delivered before the Wyoming County Medical Association, July 10, 1900.

**Published:** 1900-10

**Authors:** William Stanton

**Affiliations:** Varysburg, N. Y.


### THE PHYSICIAN.[1]

By WILLIAM STANTON, M. D., Varysburg, N. Y.

MEDICAL effort first arose from human sympathy. The friends and comrades of the sick and injured began the work, and the continuation of human suffering has constantly supplied material for those whose special fitness or generous impulses have called to this field of labor. Year upon year, and age upon age, those who have preceded us have earnestly and faithfully pursued this labor of love and mercy, gathering truth and wisdom to furnish the wonderful storehouse of knowledge to which we of today have access.

Do we appreciate our opportunities, or realise our responsibilities? Are we, each of us, doing our full duty to alleviate human suffering and add something to the sum total of medical knowledge to be left to our successors? True, we can not all lay claim to the genius which makes men inventors, but we may be able to detect errors, or bring together and put into useful form, and help assign their proper place, the discoveries of others, and thereby become only a little less useful than the discovers themselves, who often lack time, and frequently the ability to put into practical form their own inventions.

A great mistake made by many is to seek reputation at whatever cost. Now, a man may acquire a reputation, good or bad, dependent largely upon the influence, energies and volubility of his friends or enemies, but this is not success, neither is it the road to success.

The first requisite of true success is character, that wonderful structure of our own building, that quality by which we remember men when they are gone. It has been said of Gladstone "He gave us many gifts, but the most precious, and the most enduring, was himself—his character," and of his life; "he lived and labored with God before his eyes, and made a conscience of what he did."

Our profession requires of us knowledge, skill and devotion, and the first and highest aim of the physician should be to establish character. Surrounded as we are by ignorance and deception, false ideas of life and its purposes, compelled, as some of us are, to work, to plan and to economise, there are many temptations which tend to draw us from the straight and narrow way. The desire for worldy applause, the constant endeavor to surpass, the haste to acquire fame or fortune, are full of snares, but let us look these matters square in the face, and meet them bravely, as we would a contagious disease.

1. Presidential address delivered before the Wyoming County Medical Association, July 10, 1900.

We have all seen those in our own profession who, filled with a narrow, selfish ambition, seek reputation and financial success by unworthy means. We have heard their praises rise from the lips of a multitude of their henchmen. We have heard their criticisms of our professional brothers and ourselves; we have seen some of our best families join their ranks; yes, we have even paused some times and honestly asked ourselves: "does it pay to do square business, when some blatant quack or unprincipled wretch can so easily gain a following?" But just make an inventory of your losses and you will see that while you have lost a few good families, you have also lost several who frequently need a doctor dark nights and stormy days, who are always ready to take much medicine, but never quite prepared to settle your account. "Let not your heart be troubled," for even genius without character will only blaze up for a time. The reputation gained so easily will not endure.

The practitioner who makes a case of diphtheria of every follicular tonsilitis and appendicitis, of obstinate constipation, gains his notoriety at a ruinous cost, and he who strives to build for himself by backbiting his professional brethren, and refusing to meet his neighbors in council, criticises, complains and slanders, is using a weapon unsuited to the hand of an educated gentleman. It is the weapon of a pagan—a boomerang, which will surely return and reveal from whence it came.

Probably few men ever fully realise their ideal, but unless we aim high, there is no possibility of our path in life ever rising above the horizon. It is also true that only a few ever gain fame or fortune, but the hope is open to all, and as members of the grandest profession in the world, whose work is the Godgiven mission of protecting health and combating disease, thus making the lives of His creatures more certain, more happy and longer, we should honor our calling by nobleness of character, faithfulness to duty and diligence in pursuit of our ideal. And this should be the highest which is given on earth for man to follow—none other than the lowly Nazarene, who exemplified in His life and work among men what the ideal physician should embody.

No doubt some of you will say this is impractical and Utopian, but I want to tell you that what we need in our lives and work today is more of this ideal. Brought as we are face to face with the entrance and exit of souls upon the shore of time, we can not avoid the tremendous problems of life and death and must recognise an overruling providence and an unseen spiritual life. Said Professor

W. W. Keen in a recent address: "That this life with its joys, its diseases and disasters is all is denied alike by common sense, by reason and by revelation. He is the best physician who takes account of the life hereafter, as well as the life that now is, and who not only heals the body, but helps the soul." We are not called into the homes about us when health and happiness abound, but when the clouds gather, when come sickness or injury, or weariness over this world's cares, then the physician is called in, and he who can do the best work in that stricken home, is he, who, while possessing a thorough scientific knowledge is also prepared in heart and character to enter the confidence and sympathies of its inmates. Intemperance, immorality, or indecency of thought or action should never be his, but his character should be so far above reproach, and his life so like the Master, that not only those bowed with age, but the anxious mother, the young wife, coy maiden, or frightened child, may trust him in their hour of trial and so yield to him the inmost secrets of their lives; for these he must needs know, because the successful physician is he who sees and reads human nature as it is. He not only studies all the physical influences of climate, food, occupation, etc., but he also considers well, the influence which the emotions of fear, hope, anxiety and sorrow have upon the patient. There are times when the gentle touch of a friend, the calm sympathetic words of the family doctor, can give assurance to the sick, and hope to the anxious, when no medicines are of avail, and it is in this capacity that we can often carry hope and encouragement into many homes. We should keep in conscientious remembrance our duty of professional secrecy. Better suffer misconception or blame from reticence than repeat even petty matters of gossip and betray our patient.

A place where physicians sometimes betray weakness is in their relations to their brother doctors. Remember that every word of kindness and praise spoken of another practitioner, elevates the standing of the profession as a whole. The reverse is no less true. Therefore, hesitate long before expressing an unfavorable opinion of a neighbor or his work, especially from a layman's account.

The best physicians identify themselves with the organisation of their Fellows, not in name only, but in deed and in truth. I challenge you to show me one who has ever gained a worthy fame, who has not been an honored and respected, and active member of a learned guild. Our duty to our association should mean something to each of us; it should mean that we contribute something more than an occasional attendance towards its success. While serving as a member of the committee on program, I was astonished to find how indifferent men are to their opportunities. I say opportunity, and such it is, when we are asked to write a paper upon a subject of our own selection, for in this way, and this way only, for most of us, can we bring before the profession the light we have gained from personal study and observation. Medical practice is as varied as human experience, and we are each entitled to the other's deductions and conclusions. "Let your light so shine before men that they may see your good works," is the scriptural injunction. I am well aware that it requires time and effort to prepare to write upon or discuss a scientific subject understandingly, unless it be something to which we have previously—and at the pace science travels today, I may add continuously—devoted special attention, but I believe it is an unfailing rule that the greatest individual profit is always to the one who makes the preparation. The member of this association must be, indeed indolent, or impotent, or both, who has not the ability or inclination to contribute something to a cause of which he is a part.

One curse to our profession which affects its victims as the blight does a pear tree, is the twin brother of the patent medicine fiend, the presuming pharmacist, who floods us with trash for our waste basket and samples with which to pollute the waters of neighboring creeks. Some of these things are said to chase bacteria out of the hidden regions in our patients; some remove cancers, and still others organise fife and drum corps among the blood corpuscles for campaign purposes, and the formula of each is kept secret to ensure the continuance of the present "specially skilful compounding," and "avoid substitution," other pharmacists being unskilled and dishonest. This arrangement is, of course, for the benefit of the physician, and it enables him to secure it at the very reasonable price of one dollar per ounce, six ounces for five dollars, and makes the practice of medicine so easy. He can simply arrange and label the symptoms and then give the remedy advised by the aforesaid pharmacist. It requires exceptionally acute eyesight to see the difference between these proprietary articles and the ordinary patent medicine, and I believe they are all a snare and a delusion, and that he who stoops to this unscientific method and allows the other fellow to practise medicine for him is not using the intellect the Lord gave him, or the education he worked hard to get, neither is he doing his duty by his patient. Your patient employs you to treat him according to your knowledge and judgment.

I believe the profession should take a decided stand on this matter, for the use of these secret and proprietary remedies robs the doctor, deceives the patient and degrades the practice of medicine. Legitimate pharmacy is one of our best aids, but the honest compounder needs no secrecy or trade-mark any more than does the honest diagnostician.

The mistakes and fallacies of medical practice have been many. If you question this, just glance over the history of medicine and see how few remedies and methods of fifty years ago have withstood the test of time and the searching eye of scientific investigation. It often requires fine discrimination to discern between fact and fallacy, and the sifting of the wheat from the chaff is a labor of time, requiring careful and prolonged investigation; therefore, as a rule, it is well for the country physician, whose practice is forever open to criticism, to not be the first to test a new theory, but leave the experimenting to those whose cases are securely hidden in the large hospitals, where your competitor's friend never comes and the neighbors cease from troubling. But watch the reports of their results and be ready to accept truth.

The best physician is always a conservative man; he is also a careful man. Probably most individual mistakes occur from carelessness. Our work rightly claims all there is of us, if we are to succeed. We must invest our time, our energies and our money, for we can not do our best work without a proper equipment. But important as are our instruments of precision, they can in no wise replace the skilful touch, the watchful eye, the attentive ear, or the careful mind, which collects all the facts gained by the numerous avenues of investigation, and deliberately weighing them, arrives at a diagnosis and formulates a line of treatment. Do not allow your technical interest in a case to overshadow your personal interest in the patient, and put into the care of each a patience, a thoroughness, and a personal interest, such as if it were your only case, for while he likes to feel that his doctor has at his command the latest and best means of scientific investigation and is employing them for his benefit, it is also a fact, that he wants relief, and he wants it quickly.

The ever widening opportunities and necessities for scientific search and investigation, in so many and varied directions, demands of the physician a more extended knowledge and use of laboratory and technical work. But laboratory results should be given only their true place, for of two patients showing the presence of certain germs one may develop the disease, which is said to be specific, while the other does not, and again the characteristic forms of bacteria may depend as much upon the location of the growth as upon the germ itself. So to ignore the ordinary physical symptoms and trust to the laboratory alone would be, to say the least, poor judgment. On the other hand, the physician who trusts alone to the physical manifestations also ignores one of his most important diagnostic aids.

Health and disease are often spoken of as if they were things and not processes, and we are apt to overlook the fact that the body is not a thing of itself, but a wonderful organisation of living cells, each with its particular duty, and each manifesting its energy of life, by maintaining from its environments that fine balance which we call health. When this balance is disturbed, and the manifestations of cell life perverted, then we have disease, either local or general, as the case may be—not a thing but a life process as modified by its environments. Germs if present are simply factors in these abnormal processes and their detection is but one link in the chain of symptoms, indicating a certain morbid process. To be sure, we can not have tuberculosis without the tubercle bacilli, but on the other hand their presence is not tuberculosis, for they will not develop tuberculosis if placed under the bark of a tree. It requires other factors to secure their development and their destructive action. The same is true of other germs and the diseases to which their presence gives certain definite characteristics.

The early recognition of those processes, known to us as diphtheria, tuberculosis, and the like, rests largely with the microscope and the early detection of the essential and distinguishing link enables us to save untold suffering and many precious lives. If this were the only use of the microscope it would fully justify the attitude of the most successful physicians, who are depending more and more upon laboratory aid. The bacteriologist and pathologist are their frequent consultants.

It appears to me that the weak point here is that, as a rule, the man at the microscope has little or no knowledge of the case itself, and his reports are of necessity somewhat arbitrary and empirical. The two should not work independently, but the physician should be his own bacteriologist and laboratory director. He should not be satisfied to receive the report second hand, but should see for himself, then he will fully understand all the conditions, both clinical and laboratory, and be able to judge knowingly. In other words, every successful physician should have his own laboratory fully equipped for all ordinary work. I see no reason why he should not make his own examinations of cultures, urine, sputa and blood; fix, imbed, cut, stain, mount and examine microscopically his own pathological work.

As has been indicated, the detection of certain germs may in same cases confirm a diagnosis, or may render it uncertain when weighed with the other symptoms, the search for them being only one step in the study of disease. But it is a step of rapidly increasing importance and I have found that I often resort to microscopical examinations with much profit and a keen sense of satisfaction in cases where formerly I would have thought it superfluous, and I believe you will find the same satisfaction if you will bring these facilities into your own offices. But some of you will urge that you have not the time for such work. Well, if it is anything except the actual practice of medicine which is taking your time, abandon it, or else abandon medicine, and give place to some one who will do their duty by their patients. If it is an extensive practice which takes all your time, well and good, you are just in the position to take unto yourself a competent assistant, who can do some part of the work and thus become useful himself while greatly increasing yours. Furthermore, it furnishes a most pleasant and interesting diversion, not to say useful and profitable. It makes a physician less dependent and brings him into actual touch with scientific progress, and nearer to the secrets and wonders of disease. But I fancy you who have visions of greatness not in harmony with these remarks are weary and that you who will accept my ideal as yours, have already said amen.

I can think of no more fitting words in closing, none better suited to promote my ideal than those beautiful words of Gladstone: "Believe me when I tell you that the thrift of time will repay you a usuary of profit, beyond your fondest expectations, and that its waste will cause you to dwindle, mentally and spiritually, beyond your most gloomy apprehensions."

